# Efficacy of a Trial Oral Appliance in OSAS Management: A New Protocol to Recognize Responder/Nonresponder Patients

**DOI:** 10.1155/2021/8811700

**Published:** 2021-06-17

**Authors:** Marzia Segù, Alessia Cosi, Antonio Santagostini, Andrea Scribante

**Affiliations:** ^1^Department of Clinical-Surgical,Diagnostic and Paediatric Sciences, University of Pavia, Pavia, Italy; ^2^Unit of Orthodontics and Paediatric Dentistry, Section of Dentistry, Department of Clinical,Surgical,Diagnostic and Paediatric Sciences, University of Pavia, Pavia, Italy

## Abstract

Oral appliances (OAs) of various types have shown variable success in the treatment of mild-to-moderate obstructive sleep apnoea (OSA). In an OSA sample, this study evaluated the efficacy of a diagnostic trial OA (myTAP™); the efficacy of a definitive custom-fitted mandibular advancement device (MAD) (SomnoDent Flex™); and whether a trial device can be used to distinguish treatment responder from nonresponder patients. Patients underwent overnight home sleep recordings prior to and after fitting of these appliances in order to objectively assess their sleep quality in terms of polysomnographic (PSG) respiratory measures: apnoea-hypopnoea index (AHI), oxygen desaturation index (ODI), and minimum oxygen saturation (LowSpO_2_). 40 patients with symptomatic OSAS were enrolled, 33 males and 7 females, with a mean age of 55.6 ± 12.73 years and an initial (T0) AHI of 26.51 ± 14.79. Trial devices were used in 16 patients (AHI: 29.9 ± 19.97, ODI: 21.06 ± 16.05, and LowSpO_2_: 82 ± 10.22 at T0) and definitive MADs in 28 (AHI: 23.90 ± 9.19, ODI: 16.27 ± 11.34, and LowSpO_2_: 82.87 ± 6.04 at T0). Statistically significant decreases in AHI (9.59 ± 8.94, *p* < 0.0023) and ODI (8.20 ± 9.67, *p* < 0.0129) were observed after treatment with the trial device. Only 8 of the patients in the trial device group went on to use the definitive device. Treatment with the definitive MAD produced statistically significant decreases in AHI (11.46 ± 9.65, *p* < 0.0001) and ODI (9.10 ± 8.47, *p* < 0.0016) and a significant improvement in LowSpO_2_ (85.09 ± 6.86, *p* < 0.0004). Thus, both types of device proved effective in improving the PSG parameters. This study showed that introducing an easy-to-make and low-cost trial device into the therapeutic pathway of OSAS patients can circumvent the problem of individual responses to treatment by allowing effective classification of patients: in short, it allows a first distinction to be drawn between responders and nonresponders to treatment.

## 1. Introduction

Obstructive sleep apnoea syndrome (OSAS) is a disease characterized by repetitive obstruction of the upper airway during sleep. These obstructive events, due to anatomical and functional alterations of the airway, cause oxyhaemoglobin desaturation and transient repeated arousals, resulting in nonrestorative sleep [1]. Awakenings in OSAS are usually triggered by the greater respiratory effort needed to counter the phenomenon of reduced (hypopnoea) or absent (apnoea) airflow in the presence of respiratory movements. This syndrome has important systemic consequences [1–3]. Polysomnography (PSG) remains the best tool for monitoring OSAS evolution and for keeping it under control. Without a multidisciplinary approach and proper clinical follow-up, treatment of the condition is likely to fail [2].

Mandibular advancement devices (MADs) are widely recognized as the main alternative to continuous positive airway pressure (CPAP) therapy and have proven effective in OSAS patients [1, 4–7].

MADs are usually indicated for the treatment of mild-to-moderate OSAS and in subjects who do not tolerate CPAP [7]. However, several studies have found that oral devices can achieve good apnoea-hypopnoea index (AHI) reductions even in more severe OSAS cases [1, 5, 6].

In spite of promising results, it is difficult to predict which subjects could maximally benefit from MAD therapy: responses vary greatly, and MAD treatment may even seriously worsen AHI values in some patients [1, 2, 8, 9].

In the literature, trial MADs are taken into consideration because they were effective in the treatment of OSAS and are an economical alternative in selecting patients before undergoing therapy with definitive MADs [10–12].

This prospective study was conducted with the aim of exploring an alternative to the therapeutic pathway currently followed in OSAS patients. The authors propose the introduction of a test device, easy to make and inexpensive, which can be used to assess the effectiveness of mandibular advancement therapy in the individual patient. The possible advantages of such an assessment are twofold: patients with a positive response might be allowed to approach subsequent definitive MAD treatment with greater confidence, while refractory ones (patients whose symptoms worsen in the presence of the device) might be spared unnecessary expense and promptly directed to alternative treatment options.

To explore this hypothesis, as well as the possible value of a diagnostic trial device as a means of predicting responder/nonresponder patients, the present study evaluates the efficacy of a trial device and a definitive custom-fitted MAD in mild-to-severe OSAS and compares the results obtained with each.

## 2. Materials and Methods

### 2.1. Patient Selection

All the patients involved in the study were recruited through the Department of Clinical, Surgical, Diagnostic and Paediatric Sciences of the University of Pavia, Pavia, Italy, and selected by a dentist with expertise in sleep medicine on the basis of a series of criteria: medical, psychological, and dental. Individuals over the age of 18 years with PSG data and a diagnosis of mild-to-severe OSAS were eligible, as were patients who had previously refused CPAP treatment.  Table 1 shows the characteristics of the entire study cohort (PZ0) and treatment groups.

All potential participants underwent a complete history and physical examination. Those with an unsuitable oral situation (fewer than 8 teeth per arch, temporomandibular disorder, periodontitis, or other acute infections), central sleep apnoea, or severe cognitive disorders, were excluded. Pregnancy (third month of pregnancy to three months after delivery) was a further exclusion criterion.

To evaluate patient satisfaction, a detailed questionnaire collecting information about symptoms, perception of treatment efficacy, side effects (rated in terms of frequency and severity), and adherence to the treatment was administered at the end of each step of the protocol. The questionnaire also included the Epworth Sleepiness Scale (ESS) and the Berlin Questionnaire to monitor snoring, daytime sleepiness, fatigue, hypertension, and BMI variations. PSG data were analysed at baseline (T0) and after specific minimum intervals (T1, T2) with the appliance in situ. The following PSG respiratory measures were compared: AHI (representing the mean number of apnoeas and hypopnoeas per hour of sleep), the oxygen desaturation index (ODI), and minimum oxygen saturation (LowSpO_2_).

Patients read and signed an informed consent document prior to being enrolled in this study. The study was approved by the Unit Internal Review Board (17-1023).

### 2.2. Patient Groups (Table 1)

Forty patients with symptomatic OSAS met all the study criteria and were enrolled (PZ0). They were prevalently males (33 males and 7 females) and generally middle aged (mean age: 55.6 ± 12.73 years). They had an initial AHI of 26.51 ± 14.79. OSAS was mild (5 ≥ AHI < 15) in 9, moderate (15 ≥ AHI < 30) in 17, and severe (AHI ≥ 30) in 14 patients.

Sixteen patients (PZ trial group) (13 males and 3 females, mean age: 53.88 ± 12.88 years) consented to use a trial device. At T0, their respiratory variables were AHI: 29.9 ± 19.97, ODI: 21.06 ± 16.05, and LowSpO_2_: 82 ± 10.22. Four had mild (5 ≥ AHI < 15), 7 moderate (15 ≥ AHI > 30), and 5 severe (AHI ≥ 30) OSAS.

Twenty-four patients already under treatment with a definitive device at T0 served a control group. Choosing these patients as the control group allowed us to look for differences in respiratory variables between the definitive device and the trial device and therefore to verify the effectiveness of the latter.

Eight patients agreed for treatment with a definitive device after first using the trial appliance. Of these, only 4 could be included in the statistical analysis (other 4, lacking PSG data after treatment with the definitive device, were excluded).

The PZ definitive group thus comprised 28 patients (22 males and 6 females, mean age: 56.79 ± 12.33 years). Their respiratory variables at T0 were AHI: 23.90 ± 9.19, ODI: 16.27 ± 11.34, and LowSpO_2_: 82.87 ± 6.04. Five had mild (5 ≥ AHI < 15), 14 moderate (15 ≥ AHI < 30), and 9 severe (AHI ≥ 30) OSAS.

#### 2.2.1. Study Design

For patients not already under treatment with a definitive device, the protocol comprised three steps. As mentioned above, the individuals already being treated with the definitive device at T0 served as a control group allowing us to test the efficacy of the trial device.

The first step of the protocol included all the preliminary medical, dental, and neurological analyses, including the baseline (T0) PSG evaluations and administration of questionnaires. The severity of the disease was defined by the AHI, determined from the PSG data: the AHI represents the mean number of apnoeas and hypopnoeas per hour of sleep. OSAS was considered “mild” if the AHI was between 5 and 14, “moderate” if it was between 15 and 29, and “severe” if it was greater than or equal to 30.

In the second step, eligible patients not already using a definitive device were treated with a trial appliance to test their tolerance and therefore their likely response to MAD therapy. At T1, treatment with the trial device was considered effective if a new PSG study showed an AHI < 5. Patients not meeting this criterion were considered nonresponders; those who discontinued the treatment for any reason were defined nonadherent. Only responders were given the possibility to choose whether or not to proceed with treatment with a definitive MAD.

The third phase consisted of treatment with the definitive, custom-fitted device. At the end of this phase, lasting at least 6 months, PSG was repeated (T2).

Only the patients not already under treatment with a definitive device at T0 underwent all three steps of the protocol.

Constant follow-up with PSG was advised. Every step involving the use of an oral appliance lasted at least 6 months and included individual titration of the device, the administration of questionnaires, and the management of potential side effects.

Mandibular advancement titration is crucial in order to achieve the maximum therapeutic effect: it must be carried out individually and must respect the patient's physical limit. There is currently no univocal titration method: it is a trial-and-error process that must be supervised carefully by the specialist in charge to find the best treatment window [10, 11]. Accordingly, there were no device instructions to follow in advancement because activation is decided on an individual, clinical basis. In the event of muscular problems, advancement was kept gradual, symptomatic therapy was administered, and the initial trial period was extended.

#### 2.2.2. Devices

The trial appliance used in this study was myTAP™: an individually fitted two-piece mandibular advancement device and titratable. It is inexpensive compared with the definitive one and is easy to adapt to the individual needs of the single patient.

The final oral appliance chosen for this study was SomnoDent Flex™: a custom-made, two-piece mandibular advancement device with vertical extensions, titratable with a screw mechanism.

Both these devices open the airway, bringing the soft palate, tongue, and hyoid bone forward and activating the masseter and submental muscles; this action prevents the collapse of the upper airway.

An initial habituation period was envisaged during which the patient kept the device in his/her mouth for short periods of time while awake. Thereafter, the patient put in the device before going to bed and kept it in place throughout the night. Advancement was progressively activated by the dentist, not the patient.

#### 2.2.3. Statistical Analysis

The sample size (alpha = 0.05; power = 95%) for two independent study groups and a continuous primary outcome was calculated. With regard to the variable ODI (primary outcome), a mean of 16 was hypothesized, with a standard deviation of 10 [12]. The expected difference between the means was calculated as 8; therefore, each group required a minimum of 15 patients. AHI and LowSpO_2_ were considered secondary variables. Loss to follow-up and incomplete compliance with therapy were excluded.

Descriptive statistics, based on the mean, standard deviation, median, and minimum and maximum values, were calculated for all groups. The normality of the data was calculated using the Kolmogorov–Smirnov test. A paired *t*-test was used to compare AHI, ODI, and LowSpO_2_ at T0 and T1 for both devices.

For subgroup comparisons between AHI < 30 and AHI > 30 (for both devices), ANOVA and post hoc Tukey test were applied.

To evaluate the sequential effect of the trial followed by the definitive device, repeated-measures ANOVA and post hoc Tukey test were applied: T0 was taken as the baseline, while T1 and T2 represented all the index values obtained with the trial and the definitive appliances, respectively. Significance for all statistical tests was set at *p* < 0.05.

## 3. Results

### 3.1. Efficacy of myTAP™ (Tables 2 and 3)

Out of a total of 40 examined patients, 16 (PZ trial group) (13 males and 3 females; mean age: 53.88 ± 12.88 years) consented to use a trial device. Their T0 respiratory index values were AHI: 29.9 ± 19.97, ODI: 21.06 ± 16.05, and LowSpO_2_: 82 ± 10.22. Four had mild (5 ≥ AHI < 15), 7 moderate (15 ≥ AHI < 30), and 5 severe (AHI ≥ 30) OSAS. One of them, whose AHI increased from 19.6 at baseline to 34.4 at T1, was a nonresponder (drop out).

Thanks to the good outcome obtained with the trial device, 8 patients agreed to go on to therapy with a more solid device (definitive oral appliance).

**Figure 1:**
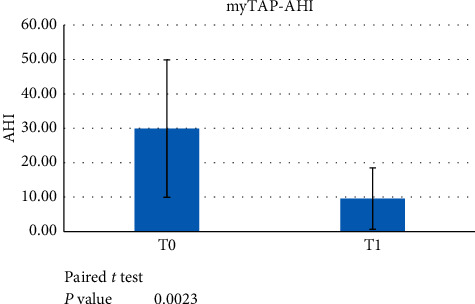
Paired *t*-test data showed a significant difference between AHI at T0 versus T1 (*p* < 0.05) when evaluating the use of the myTAP^™^ device.

**Figure 2:**
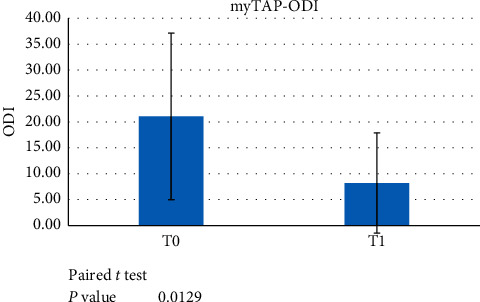
Paired *t*-test data showed a significant difference between ODI scores at T0 versus T1 (*p* < 0.05) when evaluating the use of the myTAP™ device.

**Figure 3:**
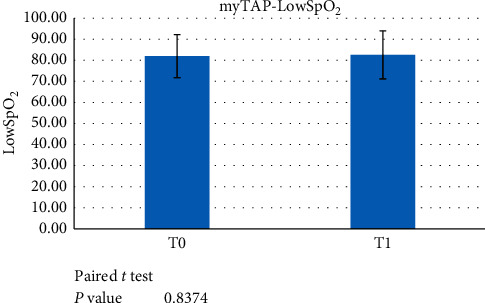
Paired *t*-test data failed to demonstrate a significant difference between LowSpO_2_ at T0 versus T1 (*p* < 0.05) when evaluating the use of the myTAP™ device.

The PZ trial group patients were analysed at T0, before the treatment started, and after a minimum 6-month treatment with the trial device (T1). A paired *t*-test showed statistically significant differences for AHI at T0 vs. T1 (Figure 1) and ODI at T0 vs. T1 (*p* < 0.05) (Figure 2). No statistically significant difference was found for LowSpO_2_ (Figure 3).

To evaluate the efficacy of the trial appliance even in the worst OSAS cases, an additional analysis comparing AHI > 30 versus AHI < 30 patients was performed.

**Figure 4:**
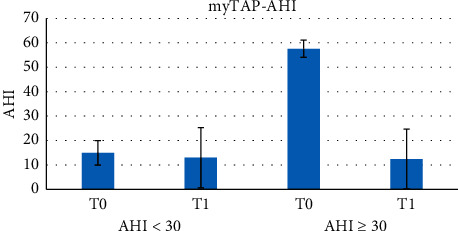
ANOVA and post hoc Tukey test showed a statistically significant difference in AHI at T0 versus T1 in the subpopulation AHI ≥ 30 (*p* < 0.05) using the myTAP™ device.

The AHI > 30 subgroup comprised 5 patients, all males, with a mean age of 47.6 ± 14.04 and an initial AHI of 56.02 ± 3.60, while the AHI < 30 subgroup was made up of 11 patients, 8 males and 3 females, with a mean age of 56.73 ± 11.88 and an initial AHI of 18.03 ± 5.28. ANOVA and post hoc Tukey test showed a statistically significant difference in AHI at T0 vs. T1 in the AHI ≥ 30 subgroup (*p* < 0.05) (Figure 4), but not in the AHI < 30 patients.

### 3.2. Efficacy of SomnoDent Flex™ (Table 2)

Of the total of 40 examined patients, 8 used SomnoDent Flex™ after first using the trial appliance. Only 4 of these, who underwent PSG after treatment with the definitive device, were included in the statistical analysis. The PZ definitive group (these four plus 24 already under treatment with a definitive device at T0) thus comprised 28 patients (22 males and 6 females, with a mean age of 56.79 ± 12.33 years). The respiratory indices at T0 were AHI: 23.90 ± 9.19, ODI: 16.27 ± 11.34, and LowSpO_2_: 82.87 ± 6.04. Five had mild (5 ≥ AHI < 15), 14 moderate (15 ≥ AHI < 30), and 9 severe (AHI ≥ 30) OSAS. Two of them (both with T0 AHI ≥ 30) were nonresponders (dropouts) as they showed an AHI increase: in one, AHI increased from 40.6 to 54.5; in the other, it increased from 37.1 to 52.1.

**Figure 5:**
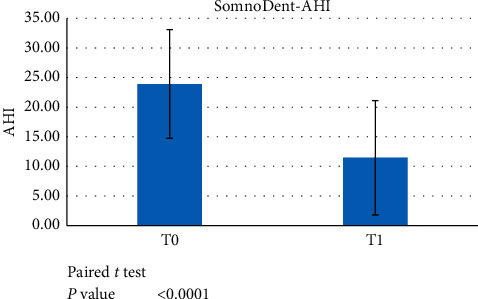
Paired *t*-test data showed significant differences between AHI at T0 versus T1 (*p* < 0.05) when evaluating the use of the SomnoDent device.

**Figure 6:**
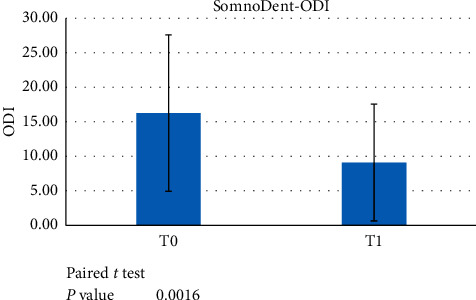
Paired *t*-test data showed a significant difference between ODI scores at T0 versus T1 (*p* < 0.05) when evaluating the use of the SomnoDent device.

**Figure 7:**
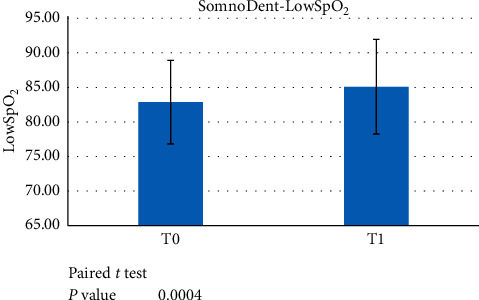
Paired *t*-test data showed a significant difference between LowSpO_2_ at T0 versus T1 (*p* < 0.05) when evaluating the use of the SomnoDent device.

This group was analysed before the treatment started and after a minimum 6-month treatment with the definitive device. A paired *t*-test showed statistically significant differences in all indexes (AHI, ODI, and LowSpO_2_) between T0 and T1 (*p* < 0.05) (Figures 5–7).

To evaluate the efficacy of the definitive appliance even in the worst OSAS cases, an additional analysis was performed comparing AHI ≥ 30 with the AHI < 30 patients in this treatment group.

**Figure 8:**
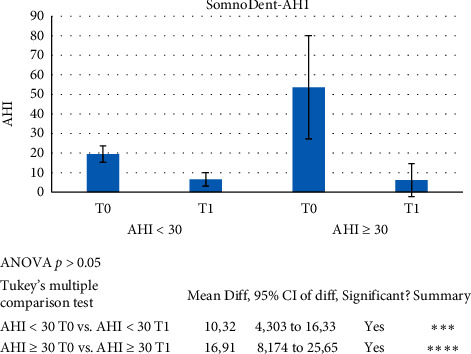
ANOVA and post hoc Tukey test showed a statistically significant difference in AHI at T0 versus T1 in the subpopulation AHI ≥ 30 (*p* < 0.05) using the SomnoDent device.

The AHI ≥ 30 subgroup comprised 9 patients, 7 males and 2 females, with a mean age of 64.33 ± 10.62 years and an initial AHI of 35.09 ± 3.83, while the AHI < 30 subgroup was made up of 19 patients, 15 males and 4 females, with a mean age of 53.16 ± 11.66 years and an initial AHI of 18.59 ± 5.28. ANOVA and post hoc Tukey test showed a statistically significant difference in AHI T0 vs. T1 in both the AHI ≥ 30 and the AHI < 30 subgroups (*p* < 0.05) (Figure 8). No other comparison showed statistical significance.

### 3.3. Evaluation of the Therapeutic Pathway from myTAP™ to SomnoDent Flex™

Of the 8 patients who used both devices, only 4 managed to undergo the T2 PSG evaluation with the definitive device in place (2 males and 2 females; mean age: 57 ± 10.92 years; AHI at T0: 20.75 ± 2.25). To evaluate the efficacy of the proposed therapeutic pathway, i.e., the sequence of the trial device (T1) followed by the definitive device (T2), a repeated-measures ANOVA and post hoc Tukey test were applied. The following variables were assessed: AHI, ODI, and LowSpO_2_.

**Figure 9:**
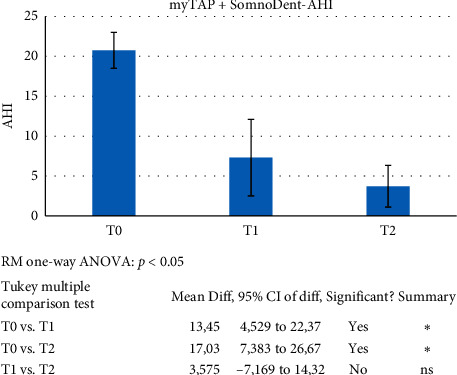
ANOVA and post hoc Tukey test were applied to evaluate the efficacy of the proposed therapeutic pathway: the trial (T1) followed by definitive device (T2). Significance for all statistical tests was set at *p* < 0.05 (C1).

**Figure 10:**
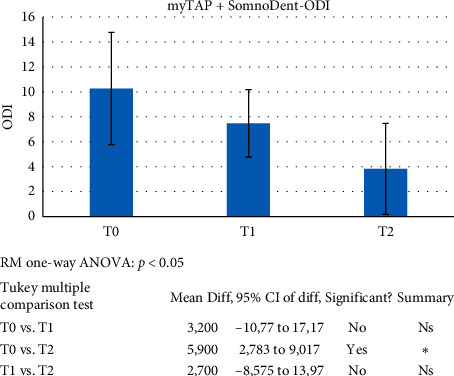
ANOVA and post hoc Tukey test were applied to evaluate the efficacy of the proposed therapeutic pathway: trial (T1) followed by definitive device (T2). Significance for all statistical tests was set at *p* < 0.05 (C2).

Significance for all statistical tests was set at *p* < 0.05. With regard to AHI, the tests revealed statistically significant differences for the T0 vs. T1 and the T0 vs. T2 comparisons. Instead, the difference between T1 and T2 was statistically irrelevant (Figure 9). ODI was found to show progressive, statistically significant differences from T0 to T2 (Figure 10). No statistical significance was found for LowSpO_2_.

In general, compliance rates were high, and patient complaints and side effects, such as dental and jaw pain, excessive salivation, and dry mouth, were minor and transient [10]: the subjects reported a notable reduction in daytime sleepiness (ESS score) and an overall improvement in their quality of life.

## 4. Discussion

The scientific community is yet to reach a consensus on the most effective protocol for distinguishing responders from nonresponders to MAD treatment.

To address this problem, the solution proposed and analysed in this study was to introduce, into the treatment plan, a trial device that is easy to make and, above all, inexpensive, in order to give patients the opportunity to ascertain their individual response to mandibular advancement treatment before committing to definitive therapy.

This approach, which was the strength of this study, allowed a more complete and accurate classification of nonresponders who, once identified, were directed to alternative types of treatment.

This clinical experience showed that most of the patients who achieved good results with the test device were willing to continue with a definitive device.

Mandibular advancement devices have the capacity to promote restorative sleep by normalising breathing during sleep; this in turn reduces daytime sleepiness and improves cognitive function, blood pressure, cardiovascular and neuropsychiatric measures, and, in general, quality of life and work performance [2–4]. Low cost, comfort, ease of use, high tolerability, and relatively mild and transient side effects of MADs allow high levels of compliance [2].

myTAP™ [13] and other trial devices [14, 15] are effective in the treatment of obstructive sleep apnoea and represent an economical alternative to customized devices. They are highly effective in selecting patients who are responders and candidates for therapy with personalized MAD.

Both devices considered in this study were able to improve the PSG indices taken into consideration:

- The trial device gave statistically significant results in terms of AHI and ODI, but no statistically significant improvement of minimum oxygen saturation (LowSpO_2_) was found
- The definitive device was effective in improving all three respiratory parameters considered (AHI, ODI, and LowSpO_2_)

Patients easily adapted to the proposed devices: side effects, which were infrequent, were transient, minor, and in no case compromised adherence to the treatment.

Interesting considerations can be drawn from the analysis of the data obtained from patients who transitioned from the test device to the final one.

In these patients, the T0 vs. T1 and T0 vs. T2 differences in AHI were both statistically significant; however, the difference between the T1 and T2 AHI was not. This latter finding can be explained by the fact that, after establishing, with the trial device, the degree of mandibular advancement corresponding to a therapeutic AHI, the definitive device is constructed with the same advancement. With regard to ODI, the only statistically significant difference was between T0 and T2: this might be considered an indication of the mechanical superiority of the definitive device over the trial one.

Although CPAP remains the gold standard for severe OSAS [4], this study, in agreement with the literature, shows that MAD therapy may produce good results even in cases of severe OSAS. Specifically, the analysed data showed the following:

- The test device was statistically effective in lowering the AHI, but showed no statistically significant data for ODI and LowSpO_2_
- The final device was statistically effective in improving AHI and ODI, but not LowSpO_2_

The finding that MAD therapy might be used to manage even the most severe forms of this disease opens a possible alternative treatment avenue for all those patients unable to tolerate CPAP. Clinical experience shows that some patients benefit from combining oral device therapy with CPAP or prove to be able to manage the disease by alternating CPAP and MAD according to the need [4].

Both appliances used in this study have intrinsic limitations, namely, the difficulty some patients have adapting to an intraoral appliance and the possibility of muscular problems related to advancement and retention problems.

This study shows that a definitive oral appliance can obtain better results than a trial one: given the material instability of the trial device, which is built with less performing materials than the final device, it is advisable to upgrade the definitive device within the first year at the latest. Otherwise, long-term retention problems may arise, especially in cases with significant activations.

Treatment failures in this setting continue to be linked to the lack of a multidisciplinary approach or proper clinical follow-up.

To ensure effective follow-up, it is worth remembering that OSAS is a chronic disease and can therefore worsen over the years; furthermore, patients' general health can also be compromised by other diseases that may affect both their OSAS and their compliance with the treatment; finally, the device itself can undergo changes (mostly due to wear), making it advisable to periodically monitor its titration and retention.

In the literature, there are no similar studies comparing the efficacy of test and definitive devices, although some articles examine the efficacy of single definitive devices [1, 16, 17] or compare two definitive devices [16, 18]; there are also studies comparing CPAP and MADs generally [19–22].

On the basis of these considerations, periodic reassessment of the device is recommended, so, too, is periodic assessment of the patient's health, in order to try to intercept medical changes or persistence of bad habits (smoking, use of alcohol, obesity, and poor sleep hygiene), reinforce compliance, and strengthen the therapeutic alliance.

The limitations of this study were the small sample size, the use of only two devices, the small number of patients who used the second device after the first, and the lack of a randomized study.

## 5. Conclusions

Individual response to treatment with oral devices varies widely in patients with OSAS, making it impossible to define a priori, which are the ideal candidates for mandibular advancement therapy.

The introduction of an easy-to-make and inexpensive trial device into the therapeutic pathway of OSAS may circumvent this problem as it seems to allow effective and individual preliminary classification of these patients as responders or nonresponders to treatment.

In short, this approach allows timely interception of nonresponder patients, who can then be directed towards alternative therapies. Responders, on the contrary, having been given the opportunity to experience the benefits of the treatment before committing to a definitive oral device, are therefore more likely to adhere to the treatment. Overall, the trial device emerges as an important clinical moment which allows the patient to begin OSAS therapeutic pathway.

The oral devices analysed in this study proved to be effective allies for the treatment of OSAS:

- The trial device (myTAP™) gave excellent results in terms of reducing PSG indices (AHI and ODI) in patients with mild and moderate OSAS. There were no statistically significant changes in LowSpO_2_. The device was also found to be effective in reducing AHI in patients with severe OSAS.
- The definitive device (SomnoDent Flex™) proved effective in improving all three parameters considered (AHI, ODI, and LowSpO_2_) in patients with mild and moderate OSAS. In patients with severe OSAS, both the AHI and the ODI showed statistically significant improvements, whereas the improvement in LowSpO_2_ was not significant.

Although the effectiveness of MADs has been widely proven, it is not yet possible to comment on the outcome of long-term therapy: at present, this approach is not supported by a single and validated protocol; its success appears to depend on the judgement and expertise of the professional and the compliance of the patient.

Although the statistical data obtained are encouraging, they need to be reinforced through further investigation of the question in larger studies.

## Figures and Tables

**Table 1:** Participants' characteristics and the devices used in each group.

	PZ0	PZ trial	PZ definitive
Number of PZ	40	16	28
Male	33	13	22
Female	7	3	6
Mean age	55.6 ± 12.73	53.88 ± 12.88	56.79 ± 12.33
AHI T0	26.51 ± 14.79	29.9 ± 19.97	AHI 23.90 ± 9.19

Number of PZ/OSAS severity			
Mild (5 ≥ AHI < 15)	9	4	5
Moderate (15 ≥ AHI < 30)	17	7	14
Severe (AHI ≥ 30)	14	5	9

**Table 2:** Short-term effects of the appliances on respiratory variables.

Appliance	Variable	Time	Mean	SD	Min	Median	Maximum
myTAP	AHI	T0	29.90	19.97	7.7	20.25	72.3
		T1	9.59	8.94	0.2	7.35	34.4
	ODI	T0	21.06	16.05	1.1	16.1	57.3
		T1	8.20	9.67	0.2	6.4	40.8
	LowSpO_2_	T0	82.00	10.22	51	85	92
		T1	82.56	11.42	55	87	92

SomnoDent	AHI	T0	23.90	9.19	8.5	21.75	40.6
		T1	11.46	9.65	0	9.95	29.9
	ODI	T0	16.27	11.34	2.9	10.6	39.5
		T1	9.10	8.47	0.2	7.45	31.1
	LowSpO_2_	T0	82.88	6.04	68	84	92
		T1	85.09	6.86	62	86	96

**Table 3:** Short-term effects of the myTAP™ device on respiratory variables.

PZ	AHI T0	AHI T1	ODI T0	ODI T1	LowSpO_2_ T0	LowSpO_2_ T1
1	7.7	3.8	39.3	40.8	51	58
2	13	6.2	37.8	6.6	85	90
3	14.4	8.7	7.7	6	83	55
4	14.4	5.8	16.1	5	87	88
5	18.7	7.4	43.5	0.7	80	91
6	19.6	34.4	13.9	8.8	88	86
7	19.7	2.4	1.1	0.3	80	80
8	19.8	11.5	21.6	6.2	90	89
9	20.7	4.1	18.4	17.1	85	88
10	23.9	12.3	57.3	0.2	82	77
11	26.4	7.3	14.7	9	72	82
12	35	12.1	18.1	2.2	80	92
13	53.8	25.1	10.4	3.9	85	76
14	58.2	11.5	5.7	7.1	92	92
15	60.8	0.6	10.3	8.6	88	92
16	72.3	0.2	8.7	8.7	85	85

## Data Availability

All the data used to support the findings of this study are available from the corresponding author upon request.
